# Machine‐learning scoring functions to improve structure‐based binding affinity prediction and virtual screening

**DOI:** 10.1002/wcms.1225

**Published:** 2015-08-28

**Authors:** Qurrat Ul Ain, Antoniya Aleksandrova, Florian D. Roessler, Pedro J. Ballester

**Affiliations:** ^1^ Department of Chemistry, Centre for Molecular InformaticsUniversity of CambridgeCambridgeUK; ^2^ Cavendish LaboratoryUniversity of CambridgeCambridgeUK; ^3^ Cancer Research Center of Marseille, (INSERM U1068, Institut Paoli‐Calmettes, Aix‐Marseille Université, CNRS UMR7258)MarseilleFrance

## Abstract

Docking tools to predict whether and how a small molecule binds to a target can be applied if a structural model of such target is available. The reliability of docking depends, however, on the accuracy of the adopted scoring function (SF). Despite intense research over the years, improving the accuracy of SFs for structure‐based binding affinity prediction or virtual screening has proven to be a challenging task for any class of method. New SFs based on modern machine‐learning regression models, which do not impose a predetermined functional form and thus are able to exploit effectively much larger amounts of experimental data, have recently been introduced. These machine‐learning SFs have been shown to outperform a wide range of classical SFs at both binding affinity prediction and virtual screening. The emerging picture from these studies is that the classical approach of using linear regression with a small number of expert‐selected structural features can be strongly improved by a machine‐learning approach based on nonlinear regression allied with comprehensive data‐driven feature selection. Furthermore, the performance of classical SFs does not grow with larger training datasets and hence this performance gap is expected to widen as more training data becomes available in the future. Other topics covered in this review include predicting the reliability of a SF on a particular target class, generating synthetic data to improve predictive performance and modeling guidelines for SF development. *WIREs Comput Mol Sci* 2015, 5:405–424. doi: 10.1002/wcms.1225

For further resources related to this article, please visit the WIREs website.

## INTRODUCTION

Docking can be applied to a range of problems such as virtual screening,^1^, ^2^, ^3^ design of screening libraries,^4^ protein‐function prediction,^5^, ^6^ or drug lead optimization^7^, ^8^ providing that a suitable structural model of the protein target is available. Operationally, the first stage of docking is pose generation, in which, the position, orientation, and conformation of a molecule as docked to the target's binding site are predicted. The second stage, called scoring, usually consists in estimating how strongly the docked pose of such putative ligand binds to the target (such strength is quantified by measures of binding affinity or free energy of binding). Whereas many relatively robust and accurate algorithms for pose generation are currently available, the inaccuracies in the prediction of binding affinity by scoring functions (SFs) continue to be the major limiting factor for the reliability of docking.^9^, ^10^ Indeed, despite intensive research over more than two decades, accurate prediction of the binding affinities for large sets of diverse protein‐ligand complexes is still one of the most important open problems in computational chemistry.

Classical SFs are classified into three groups: force field,^11^ knowledge‐based,^12^, ^13^ and empirical.^14^, ^15^ For the sake of efficiency, classical SFs do not fully account for certain physical processes that are important for molecular recognition, which in turn limits their ability to rank‐order and select small molecules by computed binding affinities. Two major limitations of SFs are their minimal description of protein flexibility and the implicit treatment of solvent. Instead of SFs, other computational methodologies based on molecular dynamics or Monte Carlo simulations can be used to model protein flexibility and desolvation upon binding. In principle, a more accurate prediction of binding affinity than that from SFs is obtained in those cases amenable to these techniques.^16^ However, such expensive free energy calculations remain impractical for the evaluation of large numbers of protein‐ligand complexes and their application is generally limited to predicting binding affinity in series of congeneric molecules binding to a single target.^17^

**Figure 1:**
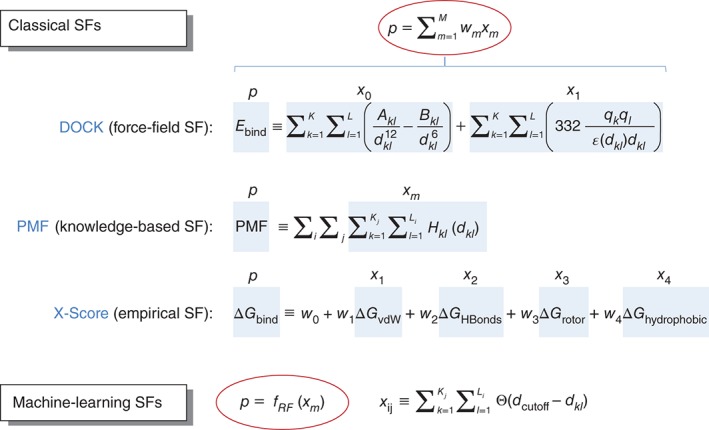
Examples of force‐field, knowledge‐based, empirical, and machine‐learning scoring functions (SFs). The first three types, collectively termed classical SFs, are distinguished by the type of structural descriptors employed. However, from a mathematical perspective, all classical SFs assume an additive functional form. By contrast, nonparametric machine‐learning SFs do not make assumptions about the form of the functional. Instead, the functional form is inferred from training data in an unbiased manner. As a result, classical and machine‐learning SFs behave very differently in practice.^20^

In addition to these two enabling simplifications, there is an important methodological issue in SF development that has received little attention until recently.^18^ Each SF assumes a predetermined theory‐inspired functional form for the relationship between the variables that characterize the complex, which may also include a set of parameters that are fitted to experimental, or simulation data, and its predicted binding affinity. Such a relationship can take the form of a sum of weighted physico‐chemical contributions to binding in the case of empirical SFs or a reverse Boltzmann methodology in the case of knowledge‐based SFs. The inherent drawback of this rigid approach is that it leads to poor predictivity in those complexes that do not conform to the modeling assumptions. As an alternative to these classical SFs, a nonparametric machine‐learning approach can be taken to capture implicitly binding interactions that are hard to model explicitly. By not imposing a particular functional form for the SF, the collective effect of intermolecular interactions in binding can be directly inferred from experimental data, which should lead to SFs with greater generality and prediction accuracy. Such an unconstrained approach was expected to result in performance improvement, as it is well known that the strong assumption of a predetermined functional form for a SF constitutes an additional source of error (e.g., imposing an additive form for the energetic contributions).^19^ This is the defining difference between machine‐learning and classical SFs: the former infers the functional form from the data, whereas the latter assumes a predetermined form that is fine‐tuned trough the estimation of its free parameters or weights from the data (Figure 1).

## WHY A REVIEW ON MACHINE‐LEARNING SFs IS TIMELY?

There are now a number of reviews on related topics endorsing the advantages and future potential of machine‐learning SFs. The first of these reviews, which praised the ability of machine‐learning SFs for effectively exploiting very large volumes of structural and interaction data, was by Huang et al.^21^ In a review of recent advances and applications of structure‐based virtual screening, Cheng et al. highlighted that a pioneering machine‐learning SF strikingly outperforms 16 state‐of‐the‐art classical SFs.^22^ Furthermore, Christoph Sotriffer argued that machine‐learning SFs are becoming increasingly popular partly due to their characteristic circumvention of the sometimes problematic modeling assumptions of classical SFs.^23^ Also, when reviewing tools for analyzing protein‐drug interactions, Lahti et al. highlighted that machine‐learning SFs improve the rank‐ordering of series of related molecules and that, as structural interatomic databases grow, machine‐learning SFs are expected to further improve.^24^ In a review dedicated to drug repurposing by structure‐based virtual screening, Ma et al. explained that machine‐learning SFs are attracting increasing attention for the estimation of protein‐ligand binding affinity.^25^ Last, Yuriev et al. noted that machine‐learning approaches in docking remain an area of active and accelerating research.^26^ Taken together, these reviews show that machine‐learning SFs are indeed a recent and fast‐growing trend.

However, no review has been devoted yet to this emerging research area, namely machine‐learning SFs where machine learning is used to replace a predetermined functional form. Different uses of machine learning in docking have been reviewed,^27^ including related applications such as iterative rescoring of poses^28^ or building optimal consensus scores.^29^, ^30^ Looking more broadly, reviews covering applications of machine learning to other research areas in drug design have also been presented.^31^, ^32^, ^33^ This review focuses instead on studies proposing machine‐learning models to improve the prediction of binding affinity, their tailored application to virtual screening (VS) and lead optimization in the context of docking as well as the presented validation exercises to assess their performance against established SFs. The rest of the article is organized as follows. Section ‘A common taxonomy for SFs’ introduces a common taxonomy for all SFs intended to rationalize method development. Section ‘Generic machine‐learning SFs to predict binding affinity’ reviews generic machine‐learning SFs to predict binding affinity for diverse protein‐ligand complexes. Section ‘Family‐specific machine‐learning SFs’ looks at the application of generic SFs and/or development of machine‐learning SFs tailored to particular protein families (target classes). Section ‘Machine‐learning SFs for virtual screening’ overviews the development and validation of machine‐learning SFs for virtual screening. Section ‘Emerging applications of machine‐learning SFs’ presents a number of emerging applications of this new class of SFs. Last, Section ‘Conclusions and future prospects’ discusses the current state and future prospects of machine‐learning SFs.

## A COMMON TAXONOMY FOR SFs

**Figure 2:**
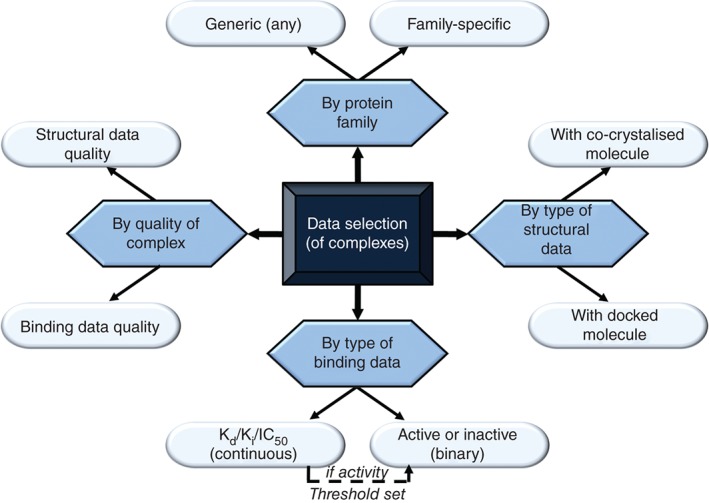
Criteria to select data to build and validate scoring functions (SFs). Protein‐ligand complexes can be selected by their quality, protein‐family membership as well as type of structural and binding data depending on intended docking application and modeling strategy. Classical SFs typically employ a few hundred x‐ray crystal structures of the highest quality along with their binding constants to score complexes with proteins from any family. In contrast, data selection for machine‐learning SFs is much more varied, with the largest training data volumes leading to the best performances.

The criteria to select training and test data mainly depend on their intended application for the SF (Figure 2). For example, SFs for binding affinity prediction need to be trained on complexes with continuous binding data, preferably binding constants. SFs for VS can also be trained on continuous binding data, but including a larger proportion of complexes with low affinity is advisable to account for the fact that screening libraries contain many more inactives than actives. Alternatively, one can select binary binding data to build a SF for VS (e.g., negative data instances can be docking poses of inactive molecules). Note that continuous data can be merged with binary data as long as the same activity threshold is adopted. Predicting continuous data will require a regression model, whereas binary data require a classifier to be built. Regardless of the application, a SF can be made to be generic by training on diverse complexes or family specific by restricting to complexes within the considered protein family.

**Figure 3:**
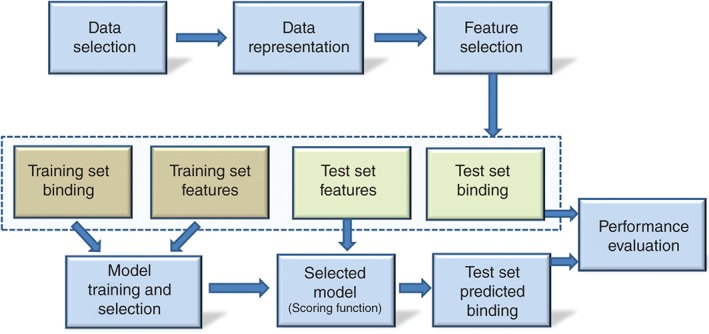
Workflow to train and validate a scoring function (SF). Feature Selection (FS) can be data‐driven or expert‐based (for simplicity, we are not representing embedded FS that would take place at the model training stage). A range of machine‐learning regression or classification models can be used for training, whereas linear regression has been used with classical SFs. Model selection has ranged from taking the best model on the training set to selecting that with the best cross‐validated performance. Metrics for model selection and performance evaluation depend on the application.

Once data are selected, a data representation is considered providing an initial set of features, which may be followed by feature selection (FS).^34^ Each complex is now represented by its values for a common set of features and a binding measurement. At this stage, complexes are usually assigned to either the training set or the test set. The training set is used for model training and selection, with the selected model after training becoming the SF. The SF can now be used to predict binding of test set complexes from their features. These predictions are ultimately compared with known measurements, which permit evaluating the performance of the SF. This process is outlined in Figure 3, with the four processed data components within the light‐blue dashed box.

There are several points to note in this process. Classical SFs are characterized by training linear models, whereas machine‐learning SFs employ nonlinear models with some form of cross‐validation in order to select that with the smallest generalization error. Note that, given the large range of machine‐learning techniques that can be used, it is highly unlikely that multiple linear regression (MLR) will obtain the best performance in every target. On the other hand, training data features determine the applicability domain^35^ of the resulting model. The binding affinities of test set complexes that are within the applicability domain should be better predicted by the model, which provides a way to identify a subset of complexes with higher accuracies than the full test set.

Benchmarks for binding affinity prediction provide a dataset suitable for constructing and validating SFs. With this purpose, one or more partitions of these data are proposed, each consisting of a training set and a test set. Examples of these benchmarks are those proposed by PDBbind^36^, ^37^ and CSAR.^38^ Here, the predictive performance of the regression‐based SF is assessed using the Pearson correlation coefficient (*R_p_*), Spearman rank‐correlation (*R_s_*), and Root Mean Square Error (RMSE) between predicted and measured binding affinity. More specifically, for a given model f, $p^{\left(n\right)}=f\left({\vec{x}}^{\left(n\right)}\right)$ is the predicted binding affinity given the features ${\vec{x}}^{\left(n\right)}$ characterizing the *n*th complex and the performance metrics are defined as:

$$\begin{array}{l} \text{RMSE}=\sqrt{\frac{1}{N}\sum_{n=1}^{N}{\left(y^{\left(n\right)}-p^{\left(n\right)}\right)}^{2}}\\ R_{p}=\frac{N\sum_{n=1}^{N}p^{\left(n\right)}y^{\left(n\right)}-\sum_{n=1}^{N}p^{\left(n\right)}\sum_{n=1}^{N}y^{\left(n\right)}}{\sqrt{\left(N\sum_{n=1}^{N}{\left(p^{\left(n\right)}\right)}^{2}-\left(\sum_{n=1}^{N}{\left(p^{\left(n\right)}\right)}^{2}\right)(N\sum_{n=1}^{N}{\left(y^{\left(n\right)}\right)}^{2}-(\sum_{n=1}^{N}{\left(y^{\left(n\right)}\right)}^{2}\right)}}\\ R_{s}=\frac{N\sum_{n=1}^{N}{p_{r}}^{\left(n\right)}{y_{r}}^{\left(n\right)}-\sum_{n=1}^{N}{p_{r}}^{\left(n\right)}\sum_{n=1}^{N}{y_{r}}^{\left(n\right)}}{\sqrt{\left(N\sum_{n=1}^{N}{\left({p_{r}}^{\left(n\right)}\right)}^{2}-\left(\sum_{n=1}^{N}{\left({p_{r}}^{\left(n\right)}\right)}^{2}\right)(N\sum_{n=1}^{N}{\left({y_{r}}^{\left(n\right)}\right)}^{2}-(\sum_{n=1}^{N}{\left({y_{r}}^{\left(n\right)}\right)}^{2}\right)}} \end{array}$$

where *N* is the number of complexes in the set, a and b are the intercept and coefficient of the linear correlation between ${\left\{p^{\left(n\right)}\right\}}_{n=1}^{N}$ and ${\left\{y^{\left(n\right)}\right\}}_{n=1}^{N}$ on the test set, whereas ${\left\{{p_{r}}^{\left(n\right)}\right\}}_{n=1}^{N}$ and ${\left\{{y_{r}}^{\left(n\right)}\right\}}_{n=1}^{N}$ are the rankings of ${\left\{p^{\left(n\right)}\right\}}_{n=1}^{N}$ and ${\left\{y^{\left(n\right)}\right\}}_{n=1}^{N}$, respectively.

On the other hand, VS aims at retrieving a subset of molecules containing the highest possible proportion of the actives contained in the screened library. SFs approach this task by ranking molecules in decreasing order of activity against the target, whether this is done using predicted binding affinity (regression) or the probability of belonging to the class of actives (classifier), and retaining the top‐ranked molecules by applying a tight cutoff to the resulting ranking. This is an early recognition problem, as only a few top‐ranking molecules can be tested in prospective applications and thus performance metrics should measure the proportion of actives that are found at the very top of the ranked list (e.g., BEDROC^39^). The enrichment factor (EF) is the most popular early recognition metric because of its simplicity and it is given by the ratio between the proportion of actives at the top x% of ranked molecules and the proportion of actives in the entire dataset (EF_x%_).

$${EF}_{x\%}=\frac{\frac{\text{ActiveMolecules}\left(x\%\right)}{\text{Molecules}\left(x\%\right)}}{\frac{\text{ActiveMolecules}\left(100\%\right)}{\text{Molecules}\left(100\%\right)}}$$

Another popular performance metric for VS is the receiver operating characteristic (ROC) curve.^40^ The ROC curve plots all the sensitivity and (1‐specificity) pairs arising from varying the cutoff from 0 to 100% of the ranked list. In this context, the sensitivity is the proportion of correctly predicted actives, whereas specificity is the proportion of correctly predicted inactives. In turn, the area under the ROC curve (ROC‐AUC), henceforth, simply referred to as AUC, measures VS performance giving equal weight to each cutoff value (AUC = 1 meaning perfect discrimination, AUC = 0.5 meaning same performance as random selection). However, in practice, only the very small cutoffs are relevant to VS performance, as only the activity of tens to hundreds of top‐ranked molecules would be experimentally tested.^41^ Indeed, despite AUC having been demonstrated to be a suboptimal measure of VS performance,^39^, ^42^, ^43^ it is widely used and thus the relative performance of reviewed SFs will often have to be assessed here on the basis of reported AUC.

Benchmarks for VS propose a test set for each considered target, each set with a group of known actives and a larger group of assumed actives (decoys). After generating the poses of these molecules as bound to the structure of the target, the SF is applied to the poses to produce a ranking of the molecules, which is in turn utilized to quantify performance through EFs or AUC. Notable examples are the directory of useful decoys (DUD)^44^ and the maximum unbiased validation (MUV)^45^ benchmarks

## GENERIC MACHINE‐LEARNING SFs TO PREDICT BINDING AFFINITY

This section focuses on the development of machine‐learning SFs to predict the binding affinity of any protein‐ligand complex. A prototypical application of generic SFs would be to identify those chemical derivatives of a known ligand that enhance their potency and selectivity against the considered target (e.g., drug lead optimization^7^, ^46^). The more accurate the ranking provided by the SF, the better guided such optimization task will be. Consequently, the SF performance at this task is measured by correlation and error metrics between predicted and binding affinities on a test dataset, as explained in the previous section.

The earliest machine‐learning SF we are aware of was introduced by Deng et al. in 2004^47^ using Kernel partial least squares (K‐PLS)^48^ as the regression model. The radial basis function (RBF) kernel is used here to carry out a transformation from the feature space to an internal representation upon which the PLS is applied. The occurrences of each protein‐ligand atom pair within a distance of 1–6 Å were used as features. Two small datasets were considered, with 61 and 105 diverse protein‐ligand complexes each. Results showed that some of the models were able to accurately predict the binding affinity of the selected test set complexes. This study constituted a proof‐of‐concept that nonlinear machine learning could be used to substitute the additive functional form of a SF. Two years later, Zhang et al.^49^ adopted k‐nearest neighbors as the regression technique and utilized a total of 517 protein‐ligand complexes from the literature. Instead of geometrical descriptors, the electronegativities of ligand and protein atom types were used as features by mapping each four neighboring atoms to one quadruplet where the corresponding atomic electronegativities were summed up. A best test set performance of *R_p_* ^2^ = 0.83 was reported.

The first study using a neural network (NN) as a generic SF was performed by Artemenko in 2008.^50^ Two different classes of descriptors were used: one class was the occurrence of atomic pairs within a certain cutoff distance and the other consisted of physico‐chemical descriptors such as van der Waals interactions, electrostatic interactions, and metal atom interactions. FS was performed by excluding highly correlated data in the training set and applying MLR to further optimize the feature set. To validate the final model, an external set built from a subset of the structures in the data was used. The best model achieved *R_p_* = 0.847 and RMSE = 1.77 on the test set. Das et al.^51^ investigated in 2010 the use of support vector machine (SVM) for regression to predict binding affinity based on the 2005 PDBbind refined set and property‐encoded shape distributions (PESD) as features. As explained by the authors, variants of the PESD‐SVM were compared to the best‐available classical SFs at that time, SFCScore,^52^ in a semiquantitative manner. They concluded that the accuracy of PESD‐SVM was similar to that of SFCScore, although slightly improved in some cases.

Looking at these pioneering studies together, it is not possible to judge whether a machine‐learning SF is more predictive than the other because the training and the test sets, which were mostly unavailable and/or not sufficiently specified, are different in each study. Furthermore, a quantitative comparison with classical SFs on the same test set is lacking, which is necessary to determine whether any of these studies improved the state‐of‐the‐art. In 2009, a comparative assessment tested 16 widely used classical SFs on the same publicly available test set, while also specifying the training set of the SF achieving the best performance (X‐Score).^36^ This benchmark was not named initially and thus, it was later referred to as the PDBbind benchmark,^18^ which provided an unambiguous and reproducible way to compare SFs on exactly the same diverse test set.

A study proposing the use of random forest (RF) to build machine‐learning SFs was published in 2010.^18^ This machine‐learning SF, RF‐Score, was the first to achieve better performance than classical SFs at predicting the binding affinity of diverse protein‐ligand complexes, hence demonstrating the potential of taking a machine‐learning approach to building generic SFs. The RF model was trained on the same 1105 complexes as X‐Score^36^ and then tested on the common test set. This RF model, called RF‐Score, obtained *R_p_* = 0.776, whereas the 16 classical SFs obtained substantially lower performance (*R_p_* ranging from 0.644 to 0.216). This study argued that the large performance improvement was due to the circumvention of the modeling assumptions that are characteristic of classical SFs (e.g., additive functional form).

Durrant and McCammon built NNScore,^53^ a NN‐based SF that combines intermolecular interaction terms from AutoDock Vina^54^ and BINANA^55^ to define a set of 17 features. The NNScore series has been largely designed for VS, an application at which these machine‐learning models excel (see Section ‘Machine‐learning SFs for virtual screening’) and thus only provided limited validation for binding affinity prediction. CScore is another NN‐based SF^56^ introducing the innovation of generating two features per each atom pair accounting for attraction and repulsion based on a distance‐dependent fuzzy membership function. On PDBbind benchmark, CScore obtained *R_p_* = 0.801, a notable improvement over RF‐Score.

More recently, Hsin et al.^57^ combined multiple docking tools and two machine‐learning systems to predict the binding affinity of redocked ligand poses. The first machine‐learning system adopted and further revised RF‐Score to include, not only intermolecular interactions, but also exploited the physico‐chemical properties of the ligand as additional features. The second machine‐learning system was used to select the three most predictive binding modes for each of the complexes. For validation, a 25‐fold cross‐validation on the PDBbind 2007 refined set was performed using an 85%‐training/15%‐test split. While this SF was calibrated with the crystal structures of training complexes, eHiTS, GOLD, and AutoDock VINA were used to produce binding modes of test complexes by redocking the ligand into the cocrystallized protein. The combination of the two machine‐learning systems obtained an average *R_p_* of 0.82 across independent folds (RF‐Score obtained an average *R_p_* of 0.60–0.64 on the same redocked poses depending on the pose generation procedure, outperforming classical SFs in all cases). This SF has not been tested yet on the PDBbind benchmark.

Regarding further applications of support vector regression (SVR), Li et al.^58^ combined SVR with knowledge‐based pairwise potentials as features (SVR‐KB), which outperformed all the classical SFs on the CSAR benchmark by a large margin. An attempt to predict enthalpy and entropy terms in addition to binding energy has also been presented,^59^ although the performance of this machine‐learning SF on the PDBbind benchmark is sensibly worse than that of other SVR‐based SFs,^56^, ^57^, ^58^ suggesting that revising the implementation of this SVR should yield better results on these terms as well. On a posterior study, Ballester^60^ introduced SVR‐Score which trained using the same data, features, and protocol as RF‐Score.^18^ On the other hand, Li et al.^61^ also used SVR as the regression model for ID‐Score. A total of 50 descriptors were chosen belonging to the following categories: atom or group interactions (van der Waals, hydrogen bonding, pi system, electrostatics, and metal‐ligand bonding interactions), energy effects (desolvation and entropic loss) and geometrical descriptors (shape matching and surface property matching). The last two SVR‐based SFs outperformed all other classical SFs, but not RF‐Score, on the PDBbind benchmark (SVR‐KB has not been tested on this set yet). It is noteworthy that SVR‐Score and ID‐Score obtained similar performance despite the far more chemically rich description of ID‐Score.

Similarly, a RF‐based SF, B2BScore,^62^ investigated the use of a more precise data representation, 131 structure‐based features (β‐contacts and crystallographic normalized B factors), but did not achieve better performance than RF‐Score on the PDBbind benchmark. In contrast, Zilian and Sotriffer^63^ presented another RF‐based SF, SFC‐Score^RF^, which outperformed RF‐Score. The only difference between both SFs was in the adopted 66 features, which included the number of rotatable bonds in the ligand, hydrogen bonds, aromatic interactions, and polar and hydrophobic contact surfaces, among others (this set of descriptors was one of the main outcomes of the industry‐academia Scoring Function Consortium or SFC^52^). The authors compared SFC‐Score^RF^ to a set of competitive classical SFs with linear PLS and MLR developed 5 years before.^52^ Importantly, this RF‐based SF strongly improved their classical counterparts from *R_s_* = 0.64 to 0.79 on the PDBbind benchmark. It is noteworthy that this very large improvement was entirely due to using a different a regression model (descriptors, training set, and test set were essentially the same).

To test the widespread assumption that more detailed features lead to better performance,^61^, ^62^, ^63^ Ballester et al.^64^ investigated the impact of the chemical description of the protein‐ligand complex on the predictive power of the resulting SF using a systematic battery of numerical experiments. Strikingly, it was found that a more precise chemical description of the complex does not generally result in a more accurate prediction of binding affinity. In particular, it was observed that binding affinity can be better predicted when calculated protonation states are not explicitly incorporated into the SF. Four factors that may be contributing to this result were discussed: error introduced by modeling assumptions, codependence of representation and regression, data restricted to the bound state, and conformational heterogeneity in data.

Overall, machine‐learning SFs have exhibited a substantial improvement over classical SFs in different binding affinity prediction benchmarks.^20^, ^57^, ^58^, ^64^, ^65^, ^66^ Furthermore, a number of studies have shown that a classical SF can easily be improved by substituting their linear regression model with nonparametric machine‐learning regression, either using RF^20^, ^63^, ^66^ or SVR.^67^ On the other hand, Ashtawy and Mahatrapa presented^68^ the first comprehensive assessment of machine‐learning SFs for binding affinity prediction, which led to training and testing 42 SFs on each training‐test data partition (six regression techniques and seven set of features). The authors observed that machine‐learning SFs using RF or boosted regression trees (BRTs) were the most frequently associated to top performance. The best performance on the PDBbind benchmark was achieved by RF::XR (*R_p_* = 0.806, *R_s_* = 0.796), which is incidentally similar to RF::VinaElem^20^ both in their sets of features and performances (*R_p_* = 0.803, *R_s_* = 0.798).

**Figure 4:**
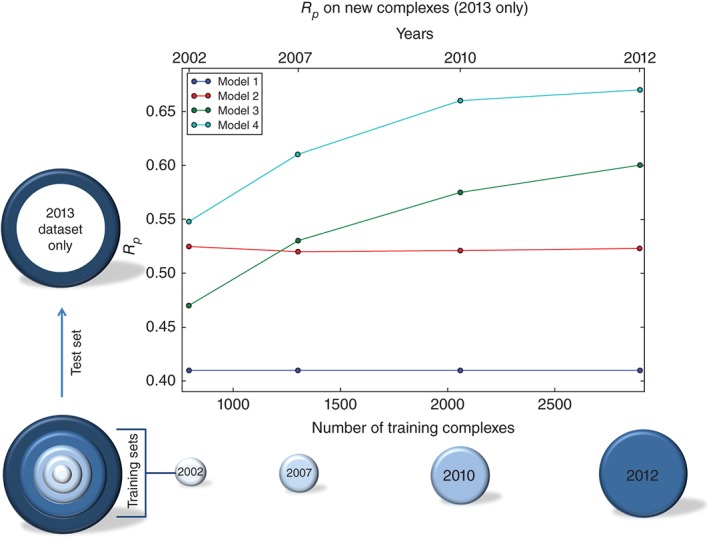
Blind test showing how test set performance (*R_p_*) grows with more training data when using random forest (models 3 and 4), but stagnates with multiple linear regression (model 2). Model 1 is AutoDock Vina acting as a baseline for performance.

Until now, we have reviewed how the combined choice of regression model and features affects the performance of the resulting SF. Recently, the future performance of these combinations was analyzed using a series of blind tests with increasingly large training sets.^20^ In the case of a classical SF, its linear regression model could not assimilate data effectively beyond a few hundred training complexes and thus its test set performance remained flat regardless of training set size. In contrast, performance of RF models on the same test set continued to increase even after having exploited 3000 training complexes (see Figure 4). This is an important result showing that more data would go wasted without producing any further improvement unless a machine‐learning approach is taken.

## FAMILY‐SPECIFIC MACHINE‐LEARNING SFs

The previous section has analyzed the performance of generic SFs on sets of diverse protein‐ligand complexes. Often, the generic SF has to be applied to complexes from a particular target and thus the question arises as to how to select the generic SF that will perform best on that target. One strategy is to take the SF that works best on a diverse test set, as it will be more likely to work better on a subset of the test set, e.g., those complexes with the target of interest, than a SF that performs worse on the full test set. Another strategy consists in evaluating the performance of the available SFs on a test set formed by complexes of the target and assuming that the best performing SF will also work best on unseen complexes of that target (i.e., complexed with different ligands). In order to have access to more test data, this analysis is usually carried out at the target class level, as grouped by global sequence similarity (loosely speaking, protein families), rather than restricted to data from a specific target within the class. A Leave‐Cluster‐Out cross‐validation (LCOCV) strategy has also been proposed, where one removes from the training set all the complexes for the target class and test the resulting SF on complexes of that target class.^69^ However, it has been shown^63^, ^70^ that LCOCV can only be interpreted as the family‐specific performance of the SF in those few target classes without any known small‐molecule binder. This section reviews studies evaluating generic SFs on a particular protein family as well as tailoring a SF to a specific family (i.e., family‐specific SFs).

A number of studies have evaluated the performance of generic machine‐learning SFs on specific protein families. Das et al.^51^ evaluated several PESD‐SVM models on a range of targets, with family‐specific performances being both above and below those obtained on the diverse test set as well as strongly target‐ and model‐dependent. Using docked rather than cocrystallized ligands in the test sets, Zilian and Sotriffer^63^ carried out a particularly careful evaluation of SFCscore^RF^ along with other classical SFs on three targets (CHK1, ERK2, and LpxC). These authors selected which SFs to submit to three blind tests based on their retrospective results on three small internal validation sets, one per target. Most of the best SFs on CHK1 and ERK2 could be anticipated following this strategy. On the other hand, the strategy of using the SF that performs best on the diverse test set, SFCscore^RF^ in this case, would have been successful on the LpxC target (SFCscore^RF^ achieved a very high correlation with measured binding affinity in this target).

Other similar studies have investigated the same question on family‐specific PDBbind test sets. Cheng et al.^36^ tested the same classical SFs on the four largest protein families in the 2007 refined set: HIV protease, trypsin, carbonic anhydrase, and thrombin. For each of these protein families, Ashtawy and Mahapatra^68^ trained a range of machine‐learning models on the 2007 refined set after having removed complexes from the considered protein family and the core set. While machine‐learning SFs outperform classical SFs on HIV protease and thrombin, the opposite was observed in the other two targets. Using the same test sets, Wang et al.^71^ investigated the family‐specific performance of the first version of RF‐Score and their RF‐based SF that combined a comprehensive set of protein sequence, binding pocket, ligand chemical structure, and intermolecular interaction features with FS, both SFs trained on the 2012 refined set without the test set complexes. Compared to previously tested SFs, Wang et al.’s SF achieved the highest performance on HIV protease, with both generic SFs providing consistently high accuracy in the other three families.

As previously argued in the context of diverse test sets,^64^ Wang et al.’s SF did not perform better than RF‐Score in every target class despite using a far more precise description of the complex. Actually, the new SF only outperformed RF‐Score on HIV protease and carbonic anhydrase,^71^ half of the evaluated target classes. The situation could be different when building family‐specific SFs, as complexes of a protein family tend to be more similar and thus more likely to be well described by a single precise characterization (e.g., adding features that are rare across all targets but common within the family such as the presence of a particular metal ion). The starting point to build family‐specific SFs is gathering the most relevant training data for the considered target class. Wang et al.^71^ also built family‐specific RF‐based SFs by exploiting target structures with their comprehensive FS protocol, which led to consistently high performance across the three more common targets in the 2012 refined set (HIV protease, trypsin, and carbonic anhydrase). Unfortunately, their generic SF was not evaluated on these three test sets as well and thus it is not possible to appreciate how the family‐specific SF compared to their generic counterpart. While training on family‐specific complexes only should reduce the complexity of the regression problem, complexes from other target classes can substantially contribute to performance on another target class.^70^ Therefore, it is still not clear whether applying a family‐specific SF is better than using a generic SF with a training set that includes all available complexes for that target.

A few more family‐specific machine‐learning SFs compiling their own datasets have been presented to date. The first of these SFs, support vector inductive logic programming (SVILP), was presented by Amini et al.,^72^ where the regression model was SVR and the features were inductive logic programming (ILP) rules. The latter are logic rules inferred from distances between predetermined fragments of the ligand and protein atoms. Over the five considered targets, it was observed that the accuracies of the classical SFs were much lower than SVILP. This study also demonstrated for the first time that docking poses can be used to train a machine‐learning SF in the absence of crystal structures to produce satisfactory results, at least in the studied target classes (HIV protease, carbonic anhydrase II, thrombin, trypsin, and factor Xa). Kinnings and colleagues followed a similar strategy by exploiting the docking poses of set of 80 InhA inhibitors along with their corresponding IC50s to construct an InhA‐specific SVR‐based SF.^67^ These authors observed that the classical SF eHiTS‐Energy SF^73^ could not predict the ranking of these molecules by IC50 (*R_s_* = 0.117). As eHiTS‐Energy returns a set 20 energy terms for each pose, 128 combinations of these terms entered a FS procedure using SVR as the wrapper. A combination of 11 eHiTS‐Energy terms with SVR led to the highest 5‐fold cross‐validation performance (*R_s_* = 0.607), hence strongly outperforming the classical eHiTS‐Energy SF at ranking the InhA inhibitors.

Regarding prospective applications of family‐specific machine‐learning SFs, Zhan et al.^74^ developed a SVR‐based SF aimed at guiding the optimization of Akt1 inhibitors. From retrospective analysis of the set of 47 Akt1 inhibitors, the authors concluded that none of the five classical SFs tested was suitable for the task. Thus, they built MD‐SVR trained on the docking poses of this set of inhibitors (the features were the five docking scores, the Akt1‐specific interaction profile and the physico‐chemical properties of the ligand). MD‐SVR was thereafter used to predict the binding affinities of a set of derivatives of a known ligand. Once the selected derivatives were synthesized and tested, the two derivatives predicted to have the highest activities were also found to have the highest measured activities (Akt1 IC50s 7nM and 17nM).

Zilian and Sotriffer^63^ achieved high predictive performance on diverse test sets without exploiting structural waters, ions, or cofactors (these were removed for all structures prior to generating the features). One might expect that explicitly including this information would improve performance further. However, the gains from including them in one target class could be outweighed by the incurred losses in other classes. In the context of family‐specific SFs, this strategy appears to be beneficial for performance, although there are surprisingly few studies addressing this particular topic. A notable exception is the work of Bortolato et al.^75^ who investigated how to incorporate solvent information into the prediction of binding affinity of adenosine receptor ligands. The authors observed that all the family‐specific linear regression models had poor performance and thus opted for a machine‐learning approach based on probabilistic classification trees^76^ with water‐related properties as features. A total of 375 ligands were divided randomly into a training set and a test set of roughly equal size. Each set had two categories, weak ligands with pKi = 6–7.5 and actives with pKi = 7.5–9. The resulting model classified the binding affinity correctly for 67% ligands in test set. It would have been interesting to compare with the same classifier without the water‐related properties in order to quantify the importance of water network maps in this target.

## MACHINE‐LEARNING SFs FOR VIRTUAL SCREENING

Another important application of machine‐learning SFs is structure‐based VS. VS is now an established family of computation techniques intended to identify bioactive molecules among a typically large set of mostly inactive molecules. As this activity is due to the molecular binding to a suitable site in the target, active molecules, or just actives, are also called binders (likewise, inactive molecules are also called inactives or nonbinders). This section reviews the application of machine‐learning SFs to VS. These SFs can be regression based to rank molecules according to predicted binding affinity (see Sections ‘Generic machine‐learning SFs to predict binding affinity’ and ‘Family‐specific machine‐learning SFs’) or classifier based to directly predict whether the molecules are binders or not. Further information about how methods for VS are trained, validated, and applied can be found in Section ‘A common taxonomy for SFs’.

An early machine‐learning classifier for VS is postDOCK,^77^ which was designed as a rescoring method to distinguish between true binders and decoys. Structural descriptors from ligand‐protein complexes and a RF classifier were used to discriminate between binding and nonbinding ligands. Fold classification on structure–structure alignment of proteins (FSSP) was applied on all known PDB complexes to cover a broad applicability of the method resulting into 152 training complexes and 44 test complexes. DOCK and ChemScore were the best ensemble model and predicted 41 binders in the test set of which 39 were actual binders. PostDOCK was applicable to diverse targets and was successful in classifying a decoy and a binder in HIV protease and thrombin but failed when applied to carbonic anhydrase. On the other hand, Das et al.^51^ applied their PESD‐SVM to VS. The experiment consisted in dividing the set of actives into three categories (strong, medium, and weak) and determining the percentage of actives that were correctly identified in each category (47, 82, and 62% respectively). While the experiment does not employ any set of inactives, it demonstrates certain ability of PESD‐SVM to retrieve actives from a given potency range.

Sato et al.^78^ investigated how different classifiers performed at VS. RF, SVM, NN, and Naïve Bayesian classifier were evaluated. The features were the counts of protein‐ligand interactions pairs such as hydrogen bonds, ionic interaction, and hydrophobic interaction (this is Pharm‐IF, an atom‐based interaction fingerprint). Classifiers were built for five target classes: PKA, SRC, carbonic anhydrase II, cathepsin K, and HIV‐1 protease. The training set was formed by ligand‐bound crystal structures, which ranged in number between 9 and 197 depending on the target class, as actives. The test set was formed by 100 actives per target from the StARlite database (currently known as ChEMBL database^79^). For each set, 2000 decoy molecules per target were selected at random from the PubChem database^80^ and assumed inactives. For those actives that were not cocrystallized with the target and all inactives, docking poses were generated using Glide. The machine‐learning classifiers based on Pharm‐IF features showed a better performance for targets with large number of complexes for training (PKA, carbonic anhydrase II, and HIV protease) and worse screening for those with few (SRC and cathepsin K). The screening efficiencies for SRC and cathepsin K were improved by adding the docking poses of their actives to the training set. In particular, EF_10%_ of the RF models were dramatically enhanced to 6.5 (SRC) and 6.3 (cathepsin K), as compared to those of the models using only the crystal structures (3.9 for SRC and 3.2 for cathepsin K). Interestingly, the enhancement was much lower when using SVM. On average across the five targets, SVM Pharm‐IF model obtained the best performance, outperforming classical SFs such as Glide and PLIF (EF_10%_ of 5.7 vs 4.2 and 4.3, respectively; 10 is the maximum possible EF_10%_).

Durrant and McCammon introduced a NN‐based classifier called NNScore,^53^ which consisted of an ensemble of NNs. These were trained on the structures of 4141 protein‐ligand complexes, where the actives and inactives were defined to have K_d_ less than or greater than 25 μM respectively. NNScore was applied to the docking poses of two small sets of molecules generated with Vina^54^ using the structures of N1 neuraminidase and *Trypanosoma brucei* RNAediting ligase I. When a small virtual screen of N1 neuraminidase was performed, the estimated EFs in the top 10 hits were 10.3 (using the average of top 24 networks) and 6.9 (using Vina). In a subsequent study, NNScore 2.0^65^ was presented using NN regression and BINANA features and Vina energetic terms as features. On the nine selected target classes, NNScore 2.0 (average AUC 0.59) was shown to outperform popular classical SFs such as AutoDock_fast_ 81 (0.51), AutoDock_rigourous_ (0.50), and Vina (0.58), except in the DHFR target (AUC_AutoDock_ 0.95, AUC_NNscore 2.0_ 0.83).

Durrant et al.^82^ also carried out a comprehensive assessment of both versions of NNScore on the DUD Benchmark.^44^ From the latter, a total of 74 known actives across the 40 DUD targets were employed. Furthermore, diverse drug‐like molecules from NCI diversity set III were used as decoys instead of using DUD decoy and docked against 40 DUD targets, resulting into 1560 NCI models. DUD decoys are chemically very similar to actives and hence tend to lack the chemical heterogeneity, which one could find in NCI diversity set. This low chemical heterogeneity may bias the evaluation of those SFs that include ligand‐only features. A total of 1634 molecules (74 actives and 1560 decoys) were docked against all 40 targets using Vina and Glide and the resulting poses were rescored with NNScore and NNScore 2.0. In this way, VS performances were obtained for each target. Results showed that the docking protocols were highly system dependent. However, on average, docking with Vina and rescoring with NNScore or NNScore 2.0 outperformed Glide docking (0.78, 0.76, and 0.73, respectively) across all 40 DUD targets, although this difference was not found to be statistically significant (*P* value = 0.16).

Kinnings et al.^83^ argued that data imbalance, in this context having many more actives than inactives, reduces the performance of machine‐learning classifiers. Thus, they proposed a multiplanar SVM classifier, which is made of *n* different SVM classifiers trained on a common set of actives and one of *n* disjoint subsets of inactives. In this way, the data imbalance problem was alleviated by having a more similar number of actives and inactives in each individual SVM, while the diversity of inactives was incorporated by making the prediction through the consensus of all balanced SVMs. The resulting SVM classifier and the classical SF eHiTS‐Energy were tested on the DUD dataset for the target of interest (INHA) and the 12 DUD datasets with the largest number of chemotypes, with former outperforming the latter in all 13 targets. Subsequently,^83^ the SVM classifier was compared to another classical SF, eHiTS‐Score (SVM regression was also included in the comparison, but no information on how this SF was trained for each DUD target was provided). From the ROC curves (no quantitative measure such as BEDROC or AUC was taken), one can see that the SVM classifier obtained the best early performance in six targets (INHA, ACHE, COX2, EGFR, FXA, and VEGFR2), whereas eHiTS‐Score performed better on four targets (ACE, CDK2, HIVRT, and SRC). In the remaining three targets, both SFs obtained similar performance (P38, PDE5, and PDGFRB).

Li et al.^84^ assessed the performance of another SVM classifier, called SVM‐SP, across 41 targets including 40 from the DUD benchmark. The entire training set comprised the crystal structures of 2018 complexes, with 135 protein‐ligand atom pairs as features. A total of 5000 decoys were docked into the structure of each target, giving rise to 41 sets of inactives. For each target, complexes with proteins homologous to the target and the corresponding 5000 inactives were used to train that family‐specific SVM‐SP. The SVM‐SP SFs consistently outperformed various classical SFs (Glide, ChemScore, GoldScore, and X‐score), with AUC values above 0.75 for five out of six groups of targets. The highest mean AUC was obtained for the group of kinases (0.83 with EGFR AUC = 0.98; FDFr‐1 AUC = 0.91). Thus, the authors used SVM‐SP to screen a small library of 1125 compounds against EGFR and CaMKII. A number of low‐micromolar cell‐active inhibitors were found for both kinases despite the inadequacy of the library, as discussed by the authors.

The same authors presented a follow‐up study^58^ where generic SVR‐based SFs were also evaluated on the same benchmark. The SVR‐EP used empirical potentials and SVR‐KB used pairwise knowledge‐based potentials from combining 17 protein and ligand SYBYL atom types. These SFs were trained on the 2010 PDBbind refined set and performed on average poorly (AUC ~ 0.52–0.55), much lower average performance than the best classical SF (Glide with AUC ~ 0.68) or the best machine‐learning SF (SVM‐SP with AUC ~ 0.80). It is noteworthy that SVR‐EP and SVR‐KB were much better at binding affinity prediction than Glide, which illustrates how important can be tailoring the SF to the intended docking application (VS here). The authors thought that a way to improve the performance of SVR‐KB was to increase the number of low affinity complexes in the training set. Hence, 1000 decoys were docked against each target and added to each training set with their pKd values set to zero (this set of SFs were called SVR‐KBD). SVR‐KBD was in this way strongly improved from SVR‐KB (AUC = 0.71 vs 0.52), now also outperforming all classical SFs, but not the SVM‐SP classifier.

Prospective VS was carried out with the first version of RF‐Score in order to search for new inhibitors of antibacterial targets (*Mycobacterium tuberculosis* and *Streptomyces coelicolor* DHQase2).^85^ A hierarchical VS strategy was followed combining ligand‐based^86^ and structure‐based techniques.^18^, ^87^ While the VS protocol involving RF‐Score performed poorly retrospectively, it obtained the best performance prospectively. Overall, this study identified 100 new DHQase inhibitors against both *M. tuberculosis* and *S. coelicolor*, which contained 48 new core scaffolds. Recently, RF‐Score has been incorporated into a user‐friendly large‐scale docking webserver^88^ to carry out prospective VS of up to 23 million purchasable molecules from the ZINC database.^89^

Ding et al.^90^ applied molecular interaction energy components (MIECs) features in combination with SVM to VS. MIECs features include van der Waals, electrostatic, hydrogen bond, desolvation energy, and the nearest distance between the target protein atoms and the ligand atoms. Two datasets with positive/active to negative/inactive ratio of 1:100 and 1:200 were compiled and the performance of MIEC‐SVM was assessed by 500 cross‐validation runs. With a very small fractions of binder in the library and <1% expectation of true positives, the average performance was very high (average MCC 0.76). Furthermore, MIEC‐SVM outperformed classical SFs (AUC 0.56 for Glide, 0.88 for X‐score and 0.99 for MIEC‐SVM in the first data sets; 0.57 for Glide, 0.89 for X‐score and 0.99 for MIEC‐SVM in the second dataset) in both above mentioned scenarios and in predicting true positives out of top 20 ligands in the 500 cross validations. The average true positives were found to be 9.8, 9.3, and 15.6 for scenario 1 (1:100) and 9.6, 6.8, and 13.0 for scenario 2 (1:200) for Glide, X‐score, and MIEC‐SVM, respectively. As MIEC‐SVM exclusively analyzed HIV protease complexes, the question arises whether MIEC‐SVM would work well in other target classes, some with a much smaller training set. To shed light into this question, training set was reduced to 100 and 50 positives against 10,000 negative samples, which led to a performance drop of just 0.2 in MCC. This experiment implies that training with a higher number of actives and inactives should result in better VS performance (Box 1).

BOX 1 **SOFTWARE IMPLEMENTING MACHINE‐LEARNING SFs**

To permit the application of these SFs and facilitate the development of further machine‐learning SFs, the following software has been released to date:

PESD‐SVM^51^: http://breneman.chem.rpi.edu/PESDSVM/

RF‐Score‐v1^18^: http://bioinformatics.oxfordjournals.org/content/suppl/2010/03/18/btq112.DC1/bioinf‐2010‐0060‐File007.zip

NNScore series^53^, ^65^, ^82^: http://nbcr.ucsd.edu/data/sw/hosted/nnscore/

RF‐Score‐v2^64^: https://bitbucket.org/aschreyer/rfscore

RF‐Score‐v3 (stand‐alone^91^): http://crcm.marseille.inserm.fr/fileadmin/rf‐score‐3.tgz

RF‐Score‐v3 (embedded in a webserver for prospective VS^88^): http://istar.cse.cuhk.edu.hk/idock

Open Drug Discovery Toolkit^92^: https://github.com/oddt/oddt

## EMERGING APPLICATIONS OF MACHINE‐LEARNING SFs

This section addresses the development of machine‐learning SFs designed to tackle applications for which there are still few studies or even none yet. An example of the latter is pose generation, where the score is used to guide the optimization of the ligand geometry so that the optimized pose is as close as possible to the experimental pose of the same ligand. The main barrier for not having yet machine‐learning SFs for pose generation is not scientific but technical. The vast majority of docking software is not open source, hence, only their developers could build and validate these fitness functions. Furthermore, these developers are experts in computational chemistry but not necessarily in machine learning, as it is suggested by the fact that no machine‐learning SFs have been developed yet by a molecular modeling software company despite their proven advantages. Moreover, the very few docking methods that are open source (e.g., Vina) are documented for users but not developers. This lack of suitable documentation makes necessary analyzing the code to figure out how to embed the new fitness function without disrupting any software functionality.

Machine‐learning SFs have been used to improve our understanding of particular molecular recognition processes. Like MLR employed by empirical SFs,^52^ some machine‐learning regression techniques can also estimate which features are more important for binding across training complexes. For example, each training set could be formed by complexes from a set of ligands bound to the same target. In this case, the importance‐based ranking of features would be in general different for sets involving different targets, which would reflect different mechanisms of binding. These studies have employed a few machine‐learning feature importance techniques to date such as RF^18^, ^63^ or ILP combined with SVM.^72^ Comparisons between different feature importance techniques applied to this problem have not been carried out yet. However, as SVM and RF can model nonlinear relationships between features in their sensitivity analysis, these are expected to be more accurate than linear approaches, which cannot account for such cooperativity effects. On the other hand, machine‐learning SFs have also been shown to be helpful for the simulation of the dynamics of molecular recognition as shown by Rupp et al.,^93^ who devised a machine‐learning SF acting as a potential energy surface to speed up molecular dynamics simulations in pharmaceutically relevant systems.

Regarding applications, linear feature importance techniques have been widely used to guide drug lead optimization. For example, when the features are intermolecular interactions, the optimization of ligand potency is often carried out by synthesizing derivatives that preserve important favorable interactions and reduce unfavorable interactions according to feature importance. Recently, a pragmatic alternative involving machine‐learning SFs has been proposed,^20^ which suggests that circumventing this interpretability stage can be an advantage in structure‐based lead optimization. This is based on the observation that extracting knowledge from a docking pose using a classical SF implies two sources of error: the accuracy of the SF itself but also how well the knowledge is extracted and used to guide the optimization. As an alternative, one could score all the derivatives directly using the machine‐learning SF to only synthesize those that are predicted to be more potent. In this way, not only prediction is likely to be more accurate but the error‐prone knowledge extraction stage is avoided.

The physics of molecular recognition processes also has the potential of improving machine‐learning SFs. However, this is a particularly challenging endeavor due to the inherent confounding factors associated to structure‐based prediction of binding. The mismatch between the respective conformations to which structural data and binding data refer to has been pointed out as one of these factors:^64^ ‘binding affinity is experimentally determined in solution along a trajectory in the codependent conformational spaces of the interacting molecules, whereas the structure represents a possible final state of that process in a crystallized environment. Consequently, very precise descriptors calculated from the structure are not necessarily more representative of the dynamics of binding than less precise descriptors’. This difficulty is also evident in practice as a number of recent SFs have been introduced with features aiming at providing a better description of the solvation effect^94^ or the hydrophobic effect,^95^ but have resulted in substantially lower predictive performance than machine‐learning SFs with far less elaborated features.^64^ Nevertheless, releasing the software calculating these features could be beneficial, as others could combine them in optimal ways with more suitable regression models and complementary features. Indeed, given the unavoidable uncertainty, rigorous and systematic numerical studies appear the most reliable way to make progress in predicting binding. In terms of less direct benefits of physics to machine‐learning SFs, molecular simulations should be useful as a way to provide homology models of targets without crystal structures, which could ultimately be used to generate diverse synthetic data with which to train machine‐learning SFs and thus extend their domain of applicability.

A machine‐learning approach appears also promising to predict protein–protein binding prediction. While there are notable differences between this problem and that of protein‐ligand binding prediction (e.g., possibility of using residue–residue features, more flexibility in the ligand protein or much less data available), many concepts and methodologies are likely to be transferable. For instance, Li et al.^96^ designed an SVR ensemble to predict the binding affinity of protein–protein complexes. This method was reported to be substantially more predictive than popular knowledge‐based SFs for protein–protein binding prediction.

Machine‐learning SFs have also been applied to the problem of experimental pose prediction (also known as native pose identification or docking power). Here, the success of a SF is evaluated by measuring the percentage of complexes for which the redocked pose top‐ranked by the SF is close to the x‐ray crystal pose (here close typically means that the root mean square deviation or RMSD between both poses is less than 2 Å). Recently, Yan and Wang have claimed^94^ that all machine‐learning SFs perform poorly at this problem because two SFs that were not designed for this problem did not perform well. Unsurprisingly, this wild generalization happens to be incorrect, as there is actually a machine‐learning SF designed for experimental pose prediction^97^ that has not only been shown to perform well at this problem, but better than seven classical SFs (GoldScore, ChemScore, ASPScore, PLPScore, GlideScore, EmodelScore, and EnergyScore) on each of four test sets employed. This is a variant of SVR, termed support vector rank regression (SVRR), which learned how to rank poses from a training set of pose pairs in which one pose is expected to be better than the other. It should be possible to use this technique to predict binding modes of complexes in challenging cases, such as those complexes where there are explicit water molecules bridging receptor and ligand, by compiling a set of such complexes and following the proposed protocol to prepare a training set.

Suitable validation practices are crucial to identify and nurture emerging applications. Therefore, it is important to make a reflection on which practices are not pointing out to productive research directions. Yan and Wang showed^94^ that their knowledge‐based SF performed better than 20 classical SFs on the update of the PDBbind benchmark,^37^ but left machine‐learning SFs out of this comparison despite performing much better on this benchmark as well (e.g., Ref 98). They justified this omission by arguing that their SF performed well at both binding affinity prediction and experimental pose prediction, whereas machine‐learning SFs do not perform well at experimental pose prediction. However, we have just seen that the latter part of their argument is a false premise. Furthermore, even if the machine‐learning SF performed poorly or was not tested for experimental pose prediction, this has no bearing whatsoever on the performance assessment of the SF for another application and hence the SF only needs to be validated for the intended application. Put in plain words, if two SFs were available, one with average performance on both binding affinity prediction and experimental pose prediction and the other with a much higher performance on binding affinity prediction but poor at experimental pose prediction: which one would you use to rank the derivatives of a ligand according to target affinity in order to identify those more potent? Obviously the latter SF, as it will provide better ranking. Moreover, if the proposed features do not lead to any sort of improved performance, then these are less suitable (i.e., worse) that other features regardless of any other consideration (e.g., their complexity^64^). On the other hand, Yuriev et al.^26^ have gone even further by claiming that no machine‐learning SF is able to match empirical SFs at VS or experimental pose prediction, again based on particular cases that were not designed to tackle these problems. Surprisingly, they cited in the same paper references demonstrating that this is actually not the case neither for experimental pose prediction^97^ nor VS,^90^ but made the claim anyway. Also, these authors considered that machine‐learning SFs are reaching their potential because there appears to be a performance plateau on the PDBbind benchmark (around 0.8 in *R*_p_). However, such analysis requires removing the homology bias at the core of this benchmark^70^ by partitioning the training and test sets randomly^66^ or chronologically^20^ in order to avoid missing the fact that the performance of these SFs actually improve with increasing training dataset size (Figure 4).

## CONCLUSIONS AND FUTURE PROSPECTS

A comprehensive overview of the studies published to date on machine‐learning SFs has just been provided. Taken together, these studies illustrate not only how vibrant this emerging research area is, but also show that there are still many open questions. This final section aims at extracting conclusions from this body of research as well as identifying the most promising avenues for future research. It is hoped that this will stimulate further progress on the development and applications of this new breed of scoring techniques in docking.

Machine‐learning SFs have consistently been found to outperform classical SFs at binding affinity prediction of diverse protein‐ligand complexes. In fact, it is possible to convert the latter into the former by simply substituting MLR with SVR or RF, a strategy that has always resulted in a more accurate SF.^20^, ^63^, ^66^, ^67^, ^68^ The availability of a common benchmark^36^ has led to an understanding of which design factors result in more accurate SFs. It is now clear that MLR is too simple to approximate the relationship between binding affinity and the feature space spanned by diverse complexes. Moreover, as more data is used for training, the predictive performance of these classical SFs stagnates, whereas the SFs based on nonparametric machine learning continue to improve.^20^, ^68^ Therefore, the availability of more complexes, whether generated in the future or privately‐held in pharmaceutical companies, will improve the accuracy of SFs as long as machine‐learning regression is employed. Regarding features (also known as energy terms or potentials), it has been shown that data‐driven FS^64^ leads to better results than expert‐based FS.^63^ Last, it is now well established that any comparison between SFs has to be based on the same nonoverlapping training and test sets, and that it is preferable for this data partition to reflect the natural diversity of the complexes we aim to predict.^20^, ^70^

Several studies have evaluated the performance of generic machine‐learning SFs on specific protein families (Section ‘Family‐specific machine‐learning SFs’). Necessarily, a SF with excellent performance on a set of diverse complexes will also excel on average across the subsets arising from partitioning this set (a set of diverse complexes is often partitioned by protein family). Thus, the larger the difference in performance of two SFs on a diverse set, the less likely will be for the worse SF to outperform the best SF on a particular protein family. This relative performance is more difficult to anticipate when the complexes to predict are not well represented in the training set. Some authors have explained the performance of a SF *a posteriori*. With this purpose, Das et al.^51^ introduced the concept of applicability domain in the prediction of binding affinity in docking, although it is commonly used in other research areas such as QSAR.^99^, ^100^, ^101^ Zilian and Sotriffer^63^ also noted that larger overlaps between training and test sets explained the target‐dependent performance of SFCscore^RF^. Being able to reliably predict how good binding affinity predictions are *a priori* would be a major advance in docking. Indeed, the availability of a reliable confidence score for each prediction will permit to obtain better performance by restricting testing to those docking poses with the highest confidence scores, as it has already been shown for QSAR, e.g., Ref 102. Another open question is whether applying a family‐specific SF is better than using a generic SF with a training set that includes all available complexes for that target, which has not been addressed yet with machine‐learning SFs. Within family‐specific approaches, there is a need for studies quantifying the benefits of including expert‐based features in a particular model against the alternative of using the same model without those features, so as to determine precisely how important these features are.

A prospective application of a family‐specific machine‐learning SF has already been carried out,^74^ leading to the discovery of several low‐nanomolar Akt1 inhibitors. It is noteworthy that this SVR model was trained on the docking poses of a set of known inhibitors, as crystal structures for these ligands were not available. This strategy has also been successfully employed with other SVR models^67^, ^72^ and RF.^57^, ^78^ Given these successes and the very large number of actives that are now known for a range of targets, training on the docking poses of these molecules should strongly increase the size of training sets and thus the performance of machine‐learning SFs. The use of these synthetic data is thus promising and there are reasons why preparing these docking poses is now timely. First, the impact of pose generation error on the prediction of binding affinity has been found to be low across many targets.^91^ Second, while the main barrier to generalize this approach is that the binding pockets of most actives are not known, there are now powerful data‐driven methods to predict whether a ligand is orthosteric or not.^103^ On the other hand, other forms of approximate data, such as low‐quality structural and binding data, have been recently shown to be beneficial for predictive performance.^98^ These studies are expected to be particularly valuable in those targets with few or no known ligand‐bound crystal structures. The key question to reflect upon here is whether the extrapolation of the SF outside its applicability domain is worse than increasing domain‐target overlap by training with approximate synthetic data.

Machine‐learning SFs have also been found to obtain better average performance than classical SFs at VS. The largest differences have been achieved by three classifiers: SVM‐SP,^58^ NNScore,^82^ and MIEC‐SVM^90^ (NNScore has the additional benefit of being released as open‐source software). In various studies,^58^, ^67^, ^82^ it has been observed that regression‐based SFs performed substantially worse than classifier‐based SFs at VS. The latter has been attributed to the low content of inactive data instances that the training sets of regression‐based SFs have, which is much higher in the training set of classifiers and screening libraries. Meroueh and coworkers demonstrated^58^ that the performance of regression‐based SFs can be boosted by increasing the content of training set inactives in each DUD target. The VS performance of the resulting regression‐based SF (SVR‐KBD) was still worse than that of the SVM‐SP classifier, but better than all classical SFs including Glide. On the other hand, training on synthetic data, whether active or inactive data instances, has also been found beneficial for VS performance.^58^, ^67^, ^78^, ^90^ However, it is still not clear how this performance varies with synthetic data size and how this variation depends on the data's ratio of actives/inactives or synthetics/crystals. There are other open questions shared with the related application of binding affinity prediction such as devising useful confidence scores on the predictions or establishing the value of data‐driven versus expert‐based FS, among others. Despite how little machine‐learning SFs are still used, there are already two successful prospective VS applications in the literature.^58^, ^85^

Regarding emerging applications, there are a few studies where machine‐learning SFs have been employed to identify which intermolecular interactions are more important for binding. The extracted knowledge can be used to guide the optimization of drug leads or chemical probes,^104^ although this optimization process can also be informed directly by a machine‐learning SF acting as a fitness function.^20^ There are also proof‐of‐concept studies showing the potential of taking a machine‐learning approach in protein–protein binding affinity prediction and experimental pose prediction, as the resulting SFs already outperform a range of classical SFs on these applications. Last, the importance of rigorous and adequate validations for identifying and nurturing emerging applications has been discussed. Without these, research efforts would have been misled away from the directions that have led to the leap in performance achieved by machine‐learning SFs.

In terms of future prospects, it is expected that new studies will shed light on the open questions outlined in this review. Also, new machine‐learning SFs with improved accuracy in the intended application will be presented. For example, many regression techniques that have not been used to build SFs yet, such as deep learning NNs,^105^ might lead to further performance improvements. On the other hand, there are many types of features that can still be investigated, from intermolecular features extracted from commercial docking softwares to freely available ligand‐only^106^ and protein‐only^107^ features, as well as any combinations thereof (the latter two types connect this research area to QSAR^108^ and PCM,^109^ respectively). It is also important that the developers of these SFs make the resulting software available to others. This will increase awareness of their accuracy and potential and permit their application to many more prospective studies. Currently, there are two freely available machine‐learning SFs that are designed to be used for lead optimization (RF‐Score‐v3^20^) and VS (NNScore^82^), respectively. Very recently, the Open Drug Discovery Toolkit (ODDT)^92^ has been released, which aims at facilitating the development and application of machine‐learning SFs as a part of computer‐aided drug discovery pipelines. Even without any further modeling advance, future training data will increase the accuracy of machine‐learning SFs for lead optimization, VS, and emerging applications on a wider range of targets and ligand chemistries.
